# Titanium(IV), Zirconium(IV), and Cerium(IV) Phosphates Synthesized Under Mild Conditions—Composition Characteristics and Evaluation of Sorption Properties Towards Copper Ions in Comparison to Commercially Available Ion-Exchange Resins

**DOI:** 10.3390/ma17246226

**Published:** 2024-12-20

**Authors:** Marta Marszałek, Marcin Piotrowski, Barbara Dziełak, Mariola Blicharz, Wiktoria Malarska, Zbigniew Wzorek

**Affiliations:** Cracow University of Technology, Faculty of Chemical Engineering and Technology, Warszawska 24, 31-155 Krakow, Poland

**Keywords:** copper sorption, titanium(IV) phosphate, zirconium(IV) phosphate, cerium(IV) phosphate, ion-exchange resin

## Abstract

The removal of copper from wastewater of mine origin requires the use of an appropriate method. Sorption methods are considered to be one of the best solutions for removing copper from industrial wastewater at low levels. Metal(IV) phosphates have been reported as excellent sorption materials that can be highly selective for copper. Therefore, the aim of this research was to synthesize titanium(IV), zirconium(IV), and cerium(IV) phosphates with a wide range of P:Metal(IV) molar ratios (0.5–10) in the reaction mixture and under mild conditions, using a simple scalable approach which requires minimal financial outlays. The obtained materials were characterized using XRD, ATR-FTIR, SEM-EDS techniques, and pH titration. To evaluate the performance of the resulting materials, their sorption properties towards copper ions were examined in comparison with selected commercially available ion-exchange resins. In each group of metal(IV) phosphates, the best material has a high ion-exchange capacity: 16.9 meq/g for titanium sorbent, 8.8 meq/g for zirconium sorbent, and 7.0 meq/g for cerium sorbent. Zirconium phosphate synthesized at a P:Zr molar ratio in the reaction mixture of 10:1 exhibits the best sorption properties towards copper ions in a solution similar to mining wastewater (acidic, saline, and containing heavy metals), better than some commercial ion-exchange resins.

## 1. Introduction

Global population growth drives demand for various types of goods, which in turn leads to an increase in the scale and intensification of industrial production. In recent decades, there has been a rise in the extraction and production of certain metals, including copper, which is important for the renewable energy transition. Copper is essential for the construction of power grids, photovoltaic installations, wind farms, and electric vehicles. Moreover, large amounts of copper are used in the construction industry (e.g., as a roofing material), for the production of electrical cables, electrical and electronic equipment, industrial machinery, and pipes for water installations [1,2,3,4,5]. The wide application of copper implies the intensive exploitation of its deposits. However, due to limited natural resources, the mining industry is struggling with the problem of using low-grade ores. Thus, obtaining high-purity copper from poor raw materials requires many treatment steps, and this generates the formation of large amounts of wastewater as well as solid waste [4,6].

One of the most important issues in the industrial production of copper is the appropriate management of water, especially the quality of discharged water [6,7,8]. Mine waters and effluents from the copper industry are generally characterized by a strong acidity, high salinity, and high content of sulfates as well as heavy metals such as Cu, Zn, Fe, Mn, Ni, Al, Cr, As, and Co, which is why they can pose a particular threat to the aquatic and terrestrial ecosystems [7,9,10]. Groundwater and surface water, as well as soils and plants, may become contaminated when untreated or improperly treated wastewater with heavy metals, including copper, enters the environment [9,11,12]. The presence of copper in rivers, even at low concentrations, is toxic to aquatic biota, especially fish, and also causes a decrease in the utility values of these rivers [12,13]. High concentrations of copper in drinking water may lead to its accumulation in human and animal bodies, and consequently to serious diseases and even deaths. The digestive and nervous systems suffer the most from elevated copper levels [6,8]. According to the guidelines of the World Health Organization (WHO) [14] as well as European Union legislation (Directive (EU) 2020/2184) [15], the maximum permissible concentration of copper in drinking water cannot exceed 2 mg/L. Soil contamination with copper results in the worsening of its physicochemical properties and a lower fertility, which consequently affects the quantity and quality of the crop obtained. In addition, copper accumulated in the soil has a toxic effect on edaphon. Excess copper in plants inhibits the growth of leaves and roots, disrupts the process of photosynthesis and nutrient uptake, and damages root cells. It should also be emphasized that by taking copper from the soil, crops include it in the food chain, which may bring about its accumulation in the human organism [16,17,18].

Due to the serious consequences for health and the environment, the removal of copper from wastewaters is a priority for both industrial effluents and mining-produced waters [5,6,7,8]. For example, in Poland, the highest permissible copper content in industrial wastewater discharged into waters or the ground is 0.5 mg/L [19]. At the same time, the recovery of copper as a critical metal is becoming attractive due to the growing demand for copper and the implementation of the circular economy model [6,20]. To remove copper from industrial wastewater, many methods can be used, such as cementation, precipitation, adsorption, membrane separation, electrodialysis, photocatalysis, and bioremediation. However, the effectiveness of the mentioned techniques varies, and the results achieved are not always satisfactory [5,8,21]. Simple methods, such as precipitation and cementation, are not sufficiently effective for low-copper-concentration ranges, while the use (on an industrial scale) of more sophisticated methods, such as membrane techniques, reverse osmosis or electrochemical methods, is difficult because of high operating costs (e.g., high electricity consumption) and relatively large investment expenditures [5,8,21]. Additionally, these technologies are more sensitive and often require additional investment to maintain their equipment performance (e.g., in the case of membrane processes, the clogging of membranes, and therefore the need for frequent replacement) [5,21]. As a result of the aforementioned, the search for simple and cost-effective methods of copper removal is ongoing. Furthermore, the selective removal of copper at lower concentrations (10–500 mg/L) from complex matrices, especially in acidic wastewater (pH < 3), is still considered a challenging task [10,20,22].

Sorption methods (adsorption and ion-exchange) are considered to be one of the best solutions for removing copper from industrial wastewater, especially acidic mine effluents. Their main advantages include their environmental friendliness, cost-effectiveness, simplicity of operation, high removal efficiency, and the possibility of adsorbent regeneration [5,6,7,8,21,22]. There are commercial solutions in this area [23,24], and in recent years, many unconventional materials have been applied or developed, including natural materials (e.g., zeolites, clays) [6,25], nanomaterials [7,26], carbon materials [27,28], biosorbents [29], and metal organic frameworks (MOFs) [30]. Despite this, new sorbents are still being searched for. At present, a great importance is attached to obtaining low-cost sorbents with a high metal-binding capacity [6]. We believe that such materials could be titanium(IV), zirconium(IV), and cerium(IV) phosphates. Metal(IV) phosphates are a group of compounds with a very diverse composition, structure, and water content, they can contain phosphate (PO_4_^3−^), hydrogen phosphate (HPO_4_^2−^), and dihydrogen phosphate (H_2_PO_4_^−^) groups [31,32,33]. Tetravalent metal phosphates can be synthesized as amorphous or crystalline materials by the reaction between phosphoric acid or phosphate salt (e.g., Na_2_HPO_4_, NaH_2_PO_4_) and metal(IV) salt (e.g., TiCl_4_, TiOSO_4_, ZrOCl_2_, ZrCl_4_, Ce(SO_4_)_2_) [34,35,36,37,38,39,40,41,42]. The most widely investigated crystalline phases are α-Ti(HPO_4_)_2_·H_2_O (α-TiP, α-titanium phosphate), γ-Ti(PO_4_)(H_2_PO_4_)·2H_2_O (γ-TiP, γ-titanium phosphate), α-Zr(HPO_4_)_2_·H_2_O (α-ZrP, α-zirconium phosphate), and γ-Zr(PO_4_)(H_2_PO_4_)·2H_2_O (γ-ZrP, γ-zirconium phosphate) [31,32]. The chemical composition and crystallinity of inorganic phosphate sorbents depend primarily on the synthesis method (including precipitation, and the hydrothermal method, sol–gel method, and reflux method), synthesis conditions (the pH, temperature, stirring speed, time of aging, etc.), and the molar ratio of the reactants [38,39,40,41].

According to the literature, both crystalline and amorphous tetravalent metal phosphates act as cation exchangers [32,34,36,37,42,43], and their ion-exchange properties result from the presence of protons in the structural hydroxyl groups, which can be exchanged for various cations [34,38,39,44]. However, many authors report that the ion-exchange capacity of these materials rests on the degree of crystallinity, and that amorphous materials exhibit better sorption properties than crystalline ones [36,37,38,44,45]. Metal(IV) phosphates are considered excellent sorption materials with a wide range of applications. They have been proven to effectively remove alkali metals [32,42,46,47], alkaline earth metals [42,48], rare earth metals [35,44,49,50,51,52], heavy metals [34,36,38,45,48,53,54,55,56,57], radionuclides [33,37,43,58,59], and dyes [60,61,62] from aqueous solutions of various compositions. Moreover, they are characterized by fast sorption kinetics, a high selectivity towards heavy metals, an insolubility in water, thermal and chemical stability, and a resistance to strongly acid and strongly alkaline media, abrasion, oxidation, and ionizing radiation, making them robust sorbents [32,34,36,38,39,40,41,42,43].

Mixed zirconium titanium phosphates have also been synthesized. These ion-exchange materials are mainly obtained by the sol–gel method [63,64,65,66], and show a similar sorption performance and similar thermal and chemical stability to single metal(IV) phosphates [63,64]. Tetravalent bimetallic phosphates have been applied for the removal of alkaline earth metals [63], rare earth metals [65], heavy metals [64], and radionuclides [66]. Wu et al. [66] demonstrated that zirconium titanium phosphate is an efficient sorbent for removing trace amounts of radioactive cesium from seawater.

The aim of this research was to synthesize titanium(IV), zirconium(IV), and cerium(IV) phosphates with a wide range of P:Metal(IV) molar ratios in the reaction mixture (0.5–10) and under mild conditions (room temperature and atmospheric pressure), to characterize them using XRD, ATR-FTIR, and SEM-EDS techniques and pH titration, as well as to examine their sorption properties towards copper ions in comparison to selected commercially available ion-exchange resins (having various surface functional groups) in order to evaluate their performance. The sorbents were synthesized not only under mild temperature and pressure conditions, but also according to a simple procedure using basic laboratory equipment and without the presence of modifying agents. This approach requires minimal financial outlays, which is an undoubted advantage of the proposed method of obtaining sorbents. The sorption properties of tetravalent metal phosphates were tested for copper ions, as some scientific reports [34,57,67,68] indicate that these materials can be highly selective towards copper. For the best sorbents and selected commercial copper-selective ion-exchange resins, sorption experiments were conducted in solutions of increasing complexity. The sorption tests were aimed at checking the suitability of the synthesized materials for the sorption of copper ions from wastewater similar to effluents from the mining industry (acidic, saline, and containing heavy metals). To the best of our knowledge, there are no studies focusing on the comparison of the sorption properties of copper ions of metal(IV) phosphates synthesized under mild conditions and copper-selective resins available on the market. We believe that our research will provide valuable information in the area of using inorganic phosphate sorbents for the removal/recovery of copper from industrial wastewater.

## 2. Materials and Methods

### 2.1. Materials

Chemicals of analytical grade were used to synthesize sorbents and test their sorption properties. The 85% phosphoric acid, 65% nitric acid, 96% sulfuric acid, cerium(IV) sulfate tetrahydrate, sodium sulfate anhydrous, potassium sulfate anhydrous, magnesium sulfate anhydrous, calcium chloride anhydrous, copper(II) sulfate pentahydrate, zinc sulfate heptahydrate, manganese(II) sulfate monohydrate, nickel(II) nitrate hexahydrate, cadmium nitrate tetrahydrate, and iron(III) nitrate nonahydrate were purchased from Chempur (Piekary Śląskie, Poland). Zirconium carbonate basic hydrate and titanium(IV) oxide sulfate sulfuric acid hydrate were provided by Alfa Aesar (Ward Hill, MA, USA).

Seven commercially available copper-selective ion-exchange resins were used as received. Puromet^MT^ MTS9100, Puromet^MT^ MTS9300, and Puromet^MT^ MTS9500 were supplied by Purolite (King of Prusia, PA, USA). AmberLite^TM^ HPR1200 H, AmberSep^TM^ M4195, and LEWATIT^®^ MonoPlus TP 260 were obtained through Sigma-Aldrich (St. Louis, MO, USA). DIAION^TM^ CR20 was purchased from Alfa Aesar (Ward Hill, MA, USA). The characteristics of the ion-exchange resins used are summarized in Table 1.

#### Solutions Used in Sorption Experiments

In the studies of sorption isotherms and kinetics, six solutions with the following Cu^2+^ concentration were used: 5 mg/L, 10 mg/L, 20 mg/L, 50 mg/L, 100 mg/L, and 200 mg/L. The solutions were prepared by dissolving an appropriate amount of CuSO_4_·5H_2_O in deionized water.

In a comparative study of copper sorption (using seven commercially available ion-exchange resins and seven phosphate sorbents), four solutions of increasing complexity (Table 2) were utilized.

### 2.2. Synthesis of Titanium(IV), Zirconium(IV), and Cerium(IV) Phosphates

All the phosphate sorbents were synthesized by the precipitation method under mild conditions, i.e., at room temperature and atmospheric pressure. Some important synthesis parameters for all materials are shown in Table 3.

#### 2.2.1. Synthesis of Titanium(IV) Phosphates

Five titanium(IV) phosphates (named as TiP1, TiP2, TiP3, TiP4, and TiP5) were synthesized. Firstly, 30 g of titanium(IV) oxide sulfate sulfuric acid hydrate was dissolved in 300 mL of 1 M H_2_SO_4_ solution, and the appropriate amount of 85% H_3_PO_4_ (allowing the assumed molar ratio of P:Ti to be achieved during the synthesis) (Table 3) was diluted in 100 mL of 1 M H_2_SO_4_ solution. Then, a clear acidic solution of TiOSO_4_ was added dropwise to the solution of H_3_PO_4_ in H_2_SO_4_ with constant stirring. After the precipitation was completed, the resulting mixture was stirred for 0.5 h, and then left for 24 h to allow the formed solid particles to age. In the next step, the precipitate was separated by centrifugation and washed with deionized water until the pH of wash water was about 4. The obtained material was dried in an oven at 80 °C for 24 h, and then ground down to the desired particle size.

#### 2.2.2. Synthesis of Zirconium(IV) Phosphates

Five zirconium(IV) phosphates (named as ZrP1, ZrP2, ZrP3, ZrP4, and ZrP5) were synthesized using an approach similar to that described previously [76]. First, a zirconium(IV) oxynitrate solution was prepared by reacting 33.3 g zirconium carbonate basic hydrate with 35 mL of 65% nitric acid. The resulting solution was then diluted with 200 mL of 1 M HNO_3_ solution. In the next step, to the diluted zirconium(IV) oxynitrate solution, an amount of 42.5% H_3_PO_4_ was gradually added with continuous stirring to obtain the assumed molar ratio of P:Zr (Table 3). After the precipitation was completed, the suspension was stirred for 0.5 h, and then allowed to stand for 24 h for the aging of the obtained precipitate. After this time, the mixture was subjected to centrifugation, and the resulting precipitate was washed with deionized water until the pH of the washings was about 4. Lastly, the material was dried in an oven at 65 °C for 60 h, and then ground down to the desired particle size.

#### 2.2.3. Synthesis of Mixed Zirconium Titanium Phosphate

Mixed zirconium titanium phosphate (named as ZrTiP) was synthesized according to a procedure similar to that reported previously [76]. First, a zirconium(IV) oxynitrate solution was prepared by reacting 20.7 g zirconium carbonate basic hydrate with 22 mL of 65% nitric acid. Next, a clear acidic solution of TiOSO_4_ was prepared by dissolving 15 g of titanium(IV) oxide sulfate sulfuric acid hydrate in 200 mL of 1 M HNO_3_ solution. The prepared solutions were mixed, and a 42.5% H_3_PO_4_ solution in a molar ratio of P:(Zr+Ti) = 2:1 was added to the resulting mixture using continuous stirring. The subsequent stages for obtaining zirconium titanium phosphate were carried out in the same way as for zirconium(IV) phosphates.

#### 2.2.4. Synthesis of Cerium(IV) Phosphates

Five cerium(IV) phosphates (named as CeP1, CeP2, CeP3, CeP4, and CeP5) were synthesized. At the beginning, 20.2 g cerium(IV) phosphate tetrahydrate was dissolved in 600 mL of 1 M H_2_SO_4_ solution. Then, to the clear acidic solution of Ce(SO_4_)_2_, a 42.5% H_3_PO_4_ solution was added dropwise, stirring constantly, in such an amount as to obtain the assumed molar ratios of P:Ce (Table 3). After the precipitation was completed, the suspension was stirred for 1.5 h, and then left for 48 h in order to contact the precipitate with the mother liquor. In the next step, the mixture was centrifuged, and the obtained precipitate was washed with deionized water until the pH of the washings was about 4. Finally, the precipitate was dried in an oven at 80 °C for 20 h, and then ground down to the desired particle size.

### 2.3. Phosphate Sorbents Characterization

The obtained phosphate sorbents were characterized using powder X-ray diffraction (XRD), attenuated total reflectance–Fourier transform infrared (ATR-FTIR) spectroscopy, and scanning electron microscopy, coupled with energy-dispersive X-ray spectrometry (SEM-EDS), and the pH titration method.

The crystallinity and phase composition of the synthesized materials were determined by a Philips X’Pert diffractometer (PANalytical, Almelo, The Netherlands) equipped with a graphite monochromator with nickel-filtered Cu-K_α_ radiation. The powder X-ray diffraction patterns were recorded in the 2θ range of 10–60°.

The identification of functional groups in the obtained metal(IV) phosphate sorbents was carried out with the use of a Nicolet iS5 FTIR spectrometer equipped with an iD7 ATR accessory heaving monolithic diamond crystal (Thermo Fisher Scientific, Waltham, MA, USA). The ATR-FTIR spectra were recorded in the range of 500–4000 cm^−1^.

The elemental composition of the synthesized sorbents was determined using an Apero 2 S LoVac high-resolution scanning electron microscope (Thermo Fisher Scientific, Waltham, MA, USA) coupled with energy-dispersive X-ray spectrometry. Analyses were performed in a point mode at several points on the sample surface.

The nature and number of exchangeable groups were determined by the direct titration of phosphate sorbents with a NaOH solution. An amount of 0.2 g of a given material was mixed with 50 mL of deionized water and titrated with 0.1 M NaOH solution. After each addition of titrant (2.0 mL), the pH of the mixture was measured using a multifunction meter CX-701 (ELMETRON, Zabrze, Poland). The titration was continued until the pH of the solution became constant. Based on the pH values before and after the exchange process, the Na^+^ uptake (meq/g) was determined.

### 2.4. Sorption Experiment in Pure Copper Ions Solutions—Study of Sorption Isotherms and Kinetics

The sorption experiment was performed at room temperature using the batch method. To start the experiment, 0.2 g of the obtained sorbent was added to 50 mL of solution with different initial copper ions concentrations (5 mg/L, 10 mg/L, 20 mg/L, 50 mg/L, 100 mg/L, and 200 mg/L) and stirred vigorously. After a specified time of contact of the sorbent with the solution (5 min, 10 min, 20 min, 50 min, 100 min, 200 min, and 72 h), a portion of the mixture was sampled, centrifuged, and the copper ions concentration in the supernatant was determined by atomic absorption spectrometry (AAS). All the experiments were performed in duplicate, and the average value was taken as the result.

The amount of copper ions sorbed by the obtained sorbents as a function of time was calculated according to Equation (1):

(1) $$q_{t}=\frac{\left(C_{0}-C_{t}\right)\cdot V}{m}$$

where *q_t_* (mg/g) is the sorption capacity at a given time, *C*_0_ (mg/L) and *C_t_* (mg/L) are the initial copper ions concentration and the concentration of copper ions at a given time, respectively, *V* (L) is the volume of copper solution (L), and *m* (g) is the mass of dry sorbent.

The amount of copper ions sorbed at equilibrium (after 72 h of contact of the sorbent with the solution) was calculated according to Equation (2):

(2) $$q_{e}=\frac{\left(C_{0}-C_{e}\right)\cdot V}{m}$$

where *q_e_* (mg/g) is the sorption capacity at equilibrium and *C_e_* (mg/L) is the copper ions concentration at equilibrium.

To analyze the sorption equilibrium, two primary sorption isotherm models (the Langmuir model and Freundlich model) were applied. The Langmuir isotherm can be represented by Equation (3):

(3) $$q_{e}=\frac{q_{m}K_{L}C_{e}}{1+K_{L}C_{e}}$$

where *q_m_* (mg/g) is the maximum sorption capacity and *K_L_* (L/mg) is the Langmuir isotherm constant. The Freundlich isotherm can be given by Equation (4):

(4) $$q_{e}=K_{F}C_{e}^{\frac{1}{n}}$$

where *K_F_* ((mg/g)·(L/mg)^1/n^) is the Freundlich isotherm constant and *n* (dimensionless) is the heterogeneity factor.

### 2.5. Sorption Experiment in Solutions of Increasing Complexity—Comparative Study

The comparative study was conducted at room temperature and according to the batch method. Typically, 0.2 g of selected sorbent or commercial ion-exchange resin was added to 50 mL of the appropriate solution and stirred vigorously. After 72 h of contact of the sorbent with the liquid phase, an aliquot of the mixture was taken, centrifuged, and the concentration of selected metal ions in the supernatant was determined by the AAS method. The sorption experiment was carried out at a pH of about 2; the pH of the mixture was adjusted using 12% H_2_SO_4_ solution. The experiment was repeated three times, and the results were averaged.

To evaluate the affinity of a given sorbent towards copper ions, the distribution coefficient *K_d_* (mL/g) values were determined according to Equation (5):

(5) $$K_{d}=\frac{C_{0}-C_{e}}{C_{e}}\cdot \frac{V}{m}$$

To assess how well copper ions are separated by a given sorbent compared to other metal ions, the separation factor *SF*_*Cu*/*Me*_ values (which represent the sorption selectivity) were calculated according to Equation (6):

(6) $${SF}_{Cu/Me}=\frac{K_{d(Cu)}}{K_{d(Me)}}$$

where *K*_*d*(*Cu*)_ (mL/g) is the distribution coefficient of copper ions and *K*_*d*(*Me*)_ (mL/g) is the distribution coefficient of selected metal ions.

### 2.6. Determination of the Concentration of Copper and Other Metals Ions

The concentrations of copper and other metals in the solutions were determined using flame injection atomic absorption spectrometry (FI-AAS) by the calibration curve method. Measurements were performed on a Perkin Elmer 1100B atomic absorption spectrometer (PerkinElmer, Waltham, MA, USA). The result of the determination was the average value of five measurements.

## 3. Results and Discussion

### 3.1. Synthesis and Characterization of Titanium(IV), Zirconium(IV), and Cerium(IV) Phosphate Sorbents

There are many reports [31,32,38,39,40,41,42,43,46,47,48,49,50,57,64,65] in the literature focusing on the synthesis of ion-exchange materials based of titanium, zirconium, and cerium phosphates. However, a lot of the synthesis methods described are expensive or difficult to scale up. Therefore, in this work, we propose the synthesis of metal(IV) phosphates in mild conditions (room temperature and atmospheric pressure), from relatively easily available raw materials (in all cases, the synthesis used raw materials available on an industrial scale, i.e., >1 MT/year), without the addition of modifying agents, and using simple equipment.

The most important parameter that determines the properties and structure of the obtained phosphates is the P:Metal(IV) molar ratio in the reaction mixture. To evaluate the influence of this parameter on the functional properties of the sorbents, syntheses were performed using different P:Metal(IV) molar ratios (0.5, 1, 2, 5, and 10). The composition of the obtained materials determined on the basis of an SEM-EDS analysis is presented in Table 4. Moreover, the composition ranges at different points were calculated as a measure of heterogeneity in sorbent composition. As can be seen, even when using a high molar ratio of P:Metal(IV) in the reaction mixture, the molar ratio of P:Metal(IV) in the solid material reaches values below 2. For a P:Metal(IV) molar ratio in the reaction mixture greater than 1, the P:Metal(IV) molar ratio in the obtained materials is in the range of 0.90–1.20 for titanium phosphates, 0.62–1.64 for zirconium phosphates, and 1.26–1.40 for cerium phosphates. Taking into account the heterogeneity of the composition at different points in the material, it can be concluded that the P:Metal(IV) molar ratio values are close to 1 (the exception is ZrP5 with a P:Metal(IV) molar ratio of 1.64) when the P:Metal(IV) molar ratio in the reaction mixture is greater than 1. The P:Metal(IV) molar ratio in the solid material for sorbents synthesized at a P:Metal(IV) molar ratio in the reaction mixture of 0.5 is close to 0.5 for TiP and ZrP sorbents (0.67 for TiP and 0.43 for ZrP sorbents), while for CeP sorbents, it is close to 1.

In the case of zirconium and titanium phosphates with a higher P:Metal(IV) molar ratio in the mixture, more homogeneous materials are obtained (lower atomic concentration ranges are observed). The homogeneity of mixed titanium zirconium phosphate is significantly lower than that of pure zirconium and titanium phosphates, so it can be assumed that the material has domains rich in titanium phosphate and zirconium phosphate and will behave like a mixture of phosphates rather than a doped material.

**Figure 1:**
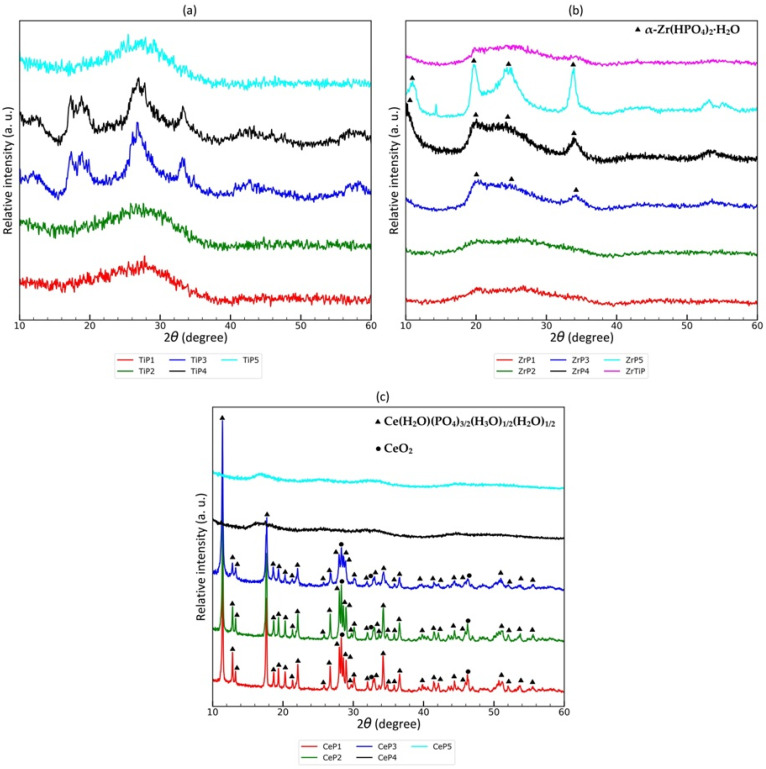
XRD patterns of: (**a**) titanium(IV) phosphate sorbents; (**b**) zirconium(IV) phosphate sorbents; and (**c**) cerium(IV) phosphate sorbents.

Figure 1 shows the X-ray diffraction patterns of the synthesized metal(IV) phosphate sorbents. Titanium phosphates are mostly completely amorphous, with only TiP3 and TiP4 containing some crystalline domains. The observed reflections (2θ = 16–20°; 2θ = 24–30°; and 2θ = 32–34°) indicate the presence of a layered structure with low crystallinity [77]. It should be noted that TiP3 and TiP4 have a P:Ti molar ratio closest to 1 throughout the series. It is likely that the proximity of the stoichiometric composition to the layered material (TiO(OH)(H_2_PO_4_)∙H_2_O) enhances the tendency to crystallize, even at room temperature. The theoretical atomic concentration for TiO(OH)(H_2_PO_4_)∙H_2_O (excluding hydrogen atoms that are not visible in the SEM-EDS spectrum) are 11% for Ti and P and 75% for O, while for TiP3, they are 10.3 atomic % for Ti, 11.5 atomic % for P, and 78.2 atomic % for O, respectively.

The tendency for crystalline domains to appear is greater in the case of zirconium phosphates than in the case of titanium phosphates. They are most visible for the ZrP5 sample. Analogous reflections are less visible for the ZrP3 and ZrP4 samples. The appearance of the X-ray diffraction patterns for the other samples (ZrP1, ZrP2, and ZrTiP) is similar. The observed reflections at 2θ = 11.55°, 2θ = 19.75°, 2θ = 24.95°, and 2θ = 33.9° indicate the presence of the α-Zr(HPO_4_)_2_∙H_2_O phase (space group: P2_1_/c, PDF-2 reference number: 01-071-1529). The high phosphoric-acid-to-titanium-ions molar ratio as well as low pH during the precipitation process favor the formation of the crystalline phase, even at room temperature.

Two amorphous and three fully crystalline materials were obtained as a result of the precipitation of cerium ions using phosphoric acid. Crystalline materials were obtained at a P:Ce molar ratio less than or equal to 2. At higher ratios, amorphous materials were formed. The crystalline phase was identified as Ce(H_2_O)(PO_4_)_3/2_(H_3_O)_1/2_(H_2_O)_1/2_ (space group: C12/c1, PDF-2 reference number: 01-077-5163), which was contaminated with cerium dioxide for samples synthesized at P:Ce molar ratios of 0.5 and 1. The results of the comparison of the theoretical composition with SEM-EDS for the assigned phase also indicate a correct identification; however, the Ce and P concentrations slightly higher than the theoretical concentrations suggest a lower content of crystal water (or the presence of CeO_2_) in the samples (the theoretical atomic % of Ce is 9.5, while from the SEM-EDS analysis, it is 11.2–12.4; the theoretical atomic % of P is 14.3, while from the SEM-EDS analysis, it is 14.1–16.2; and the theoretical atomic % of O is 76.2, while from the SEM-EDS analysis, it is 71.4–74.7).

According to the scientific report [78], a variety of crystalline structures were synthesized based on cerium(IV) phosphate. It turned out that the cerium(IV) phosphate system is not similar to the analogous systems with zirconium and titanium phosphates. Despite the efforts, the structures obtained did not correspond to the alpha or gamma structures for zirconium and titanium phosphates. According to the literature [78], during the precipitation of cerium(IV) phosphate in a nitric acid solution using phosphoric acid at an elevated temperature, a tunnel 3D framework structure with the stoichiometric formula Ce(H_2_O)(PO_4_)_3/2_(H_3_O)_1/2_(H_2_O)_1/2_ is formed.

**Figure 2:**
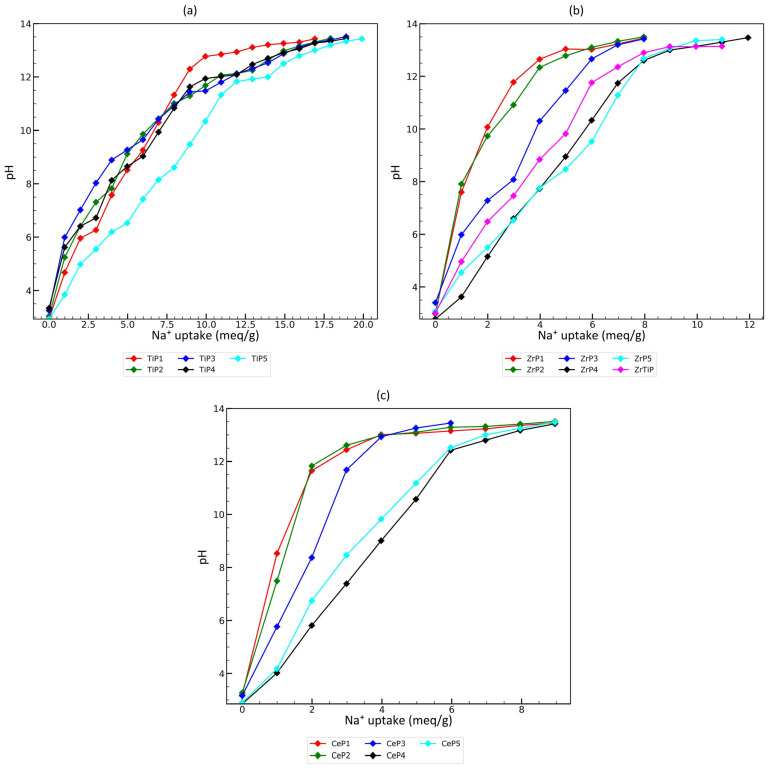
Titration curves of: (**a**) titanium(IV) phosphate sorbents; (**b**) zirconium(IV) phosphate sorbents; and (**c**) cerium(IV) phosphate sorbents.

The determination of the total ion-exchange capacity was performed by titrating the material suspension with 0.1 M NaOH. The results are presented in Table 5 and the pH-NaOH equivalent is given in Figure 2. Moreover, in the case of highly homogeneous materials, the formulas illustrating the obtained materials were proposed based on the results of SEM-EDS analyses and titrations.

Titanium phosphates exhibit a sodium ion capacity in the range of 12.3–16.9 meq/g, which is better than in the case of cerium and zirconium materials. The obtained values of the sodium ion-exchange capacity are higher than those reported in the literature for amorphous titanium phosphates, i.e., 3.1 meq/g [45] and 5.9–7.2 meq/g [77]. According to the calculation, oxide groups, hydroxide groups, and hydrogen phosphate groups should be present in the structure of the obtained materials. Sorbents synthesized at different P:Metal(IV) molar ratios exhibit different ratios of these functional groups. The titration curves (Figure 2a) are typical for amorphous materials.

Zirconium phosphates exhibit a sodium ion capacity in the range of 4.9–8.8 meq/g, which is a better result than in the case of cerium materials. The obtained values of the sodium ion-exchange capacity are higher than those reported in the literature for amorphous zirconium phosphates, e.g., the sodium ion-exchange capacity determined at pH 10 by Jiang et al. [36] and Pan et al. [55] was about 3 meq/g, while in this study, it is 6.2 meq/g for the best material (ZrP5). The structure of the obtained sorbents cannot be explained by the pure alpha zirconium phosphate structure (α-Zr(HPO_4_)_2_∙H_2_O). The calculations based on SEM-EDS and titration data indicate that both hydroxyl and oxide groups should be present in the material. The titration curves (Figure 2b) are typical for amorphous materials.

Cerium materials exhibit a sodium ion-exchange capacity in the range of 3.8–7.5 meq/g. Amorphous CeP4 and CeP5 show a more than one-and-a-half-times higher ion-exchange capacity than crystalline samples CeP1, CeP2, and CeP3. Comparing the ion-exchange capacity for CeP4 and CeP5, it can be concluded that the P:Ce molar ratio used in the synthesis had no effect on the ion-exchange capacity. The calculated theoretical ion-exchange capacity for Ce(H_2_O)(PO_4_)_3/2_(H_3_O)_1/2_(H_2_O)_1/2_ phase (which, according to the XRD results, is present in the CeP1 sample) is 1.57 meq Na^+^/g, while the determined capacity is more than two times higher. This may suggest that other phases (e.g., the amorphous phase) are present in the sorbent sample. It is probable that not only hydrogen phosphate groups are present in the samples, but also oxides (which was proven by the XRD results). It should be noted that during the precipitation of phosphate, the hydrolysis of cerium ions may occur. The titration curves (Figure 2c) are typical for amorphous materials.

To sum up, the obtained values of the ion-exchange capacity for zirconium and especially titanium materials are much higher than the maximum capacities of typical ion-exchange resins (the typical maximum capacity is ≤2.5 eq/L ≈ 3.2 meq/g). Therefore, the synthesized amorphous titanium and zirconium phosphates may be an interesting substitute for general-purpose ion-exchange resins. All titration curves are typical for amorphous materials—there are no clear, well-separated steps (S-shaped curves), associated with the neutralization of individual functional groups with different acid-base properties (different pK_a_). However, in several cases (e.g., ZrP3, TiP4, and TiP5), there are some deviations present from the straight line, which may be related to the presence of significantly different phosphate groups. Perhaps performing a titration with a higher resolution and deconvolution of the curve would answer the question of whether there are functional groups with different pK_a_, but such a study was out of the scope of this article.

Based on the calculated formula of the obtained materials (in cases where the materials were homogeneous), some general conclusions may be drawn:

- The obtained materials contain different amounts of oxide, hydroxide, phosphate, hydrogen phosphate, and dihydrogen phosphate moieties, and the relative concentration of these groups increases with the P:Metal(IV) molar ratio, as in the following series: H_2_PO_4_^−^ > HPO_4_^2−^ > PO_4_^3−^ ≈ OH^−^≈O^2−^ (at the highest P:Metal(IV) molar ratio, the highest concentration of H_2_PO_4_^−^ is achieved);
- In most cases, the materials are hydrated;
- In order to explain the achieved sorption capacity of Na^+^ ions, the participation of OH^−^ groups in the process must be taken into account.

We are convinced that in many other studies reported in the literature concerning amorphous metal(IV) phosphates, there is a similar relationship, i.e., small changes in the synthesis conditions cause continuous changes in the composition and structure of the obtained materials.

**Figure 3:**
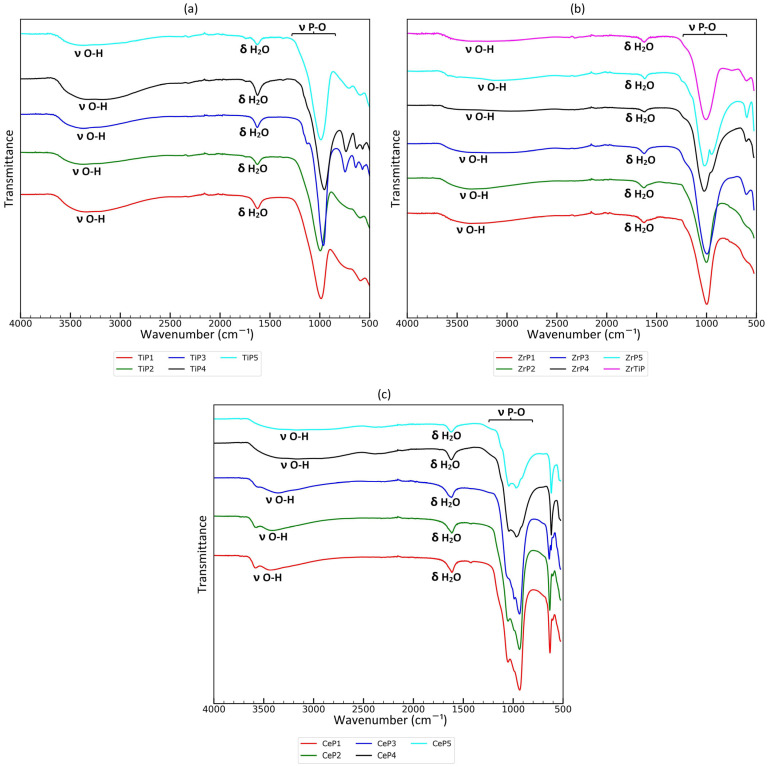
ATR-FTIR spectra of: (**a**) titanium(IV) phosphate sorbents; (**b**) zirconium(IV) phosphate sorbents; and (**c**) cerium(IV) phosphate sorbents.

In order to better understand the composition of the surface groups of the synthesized sorbents, ATR-FTIR analyses were performed. The ATR-FTIR spectra of obtained titanium(IV), zirconium(IV), and cerium(IV) phosphates are shown in Figure 3. In the case of titanium phosphates, moderately intensive wide and complex bands in the stretching of the hydroxyl group (O-H) region (2600–3700 cm^−1^) are observed in all the samples. The intensity of these bands does not correspond to the P:Ti molar ratio in the reaction mixture, e.g., the least intense bands are observed for TiP2 and TiP5 materials, and the most intense for TiP4. A band corresponding to water present in the sample (about 1640 cm^−1^) is observed for all samples. Two shoulder bands with maxima at 1130 and 1160 cm^−1^ are present in TiP3 and TiP4 materials, which contain crystal domains. Such bands are not observed in fully amorphous materials. According to the literature, shoulder bands are typically attributed to antisymmetric ν(P-O) vibrations [79], but their occurrence was observed at slightly higher wavenumbers. The main bands with a maximum at 955–995 cm^−1^ are also different for fully amorphous and crystal domain-containing samples. For fully amorphous materials, such bands have a maximum at 987–995 cm^−1^, while for TiP3 and TiP4 samples it is shifted to lower wavenumbers (955–964 cm^−1^). These bands are attributed to the symmetrical stretching of P-O and antisymmetric stretching of P-OH groups in O_2_P(OH)_2_ units, respectively [79]. Also, in the lower wavenumber region (400–900 cm^−1^), the spectra for TiP3 and TiP4 are distinctly different from the others. In the case of TiP3 and TiP4, five sharp bands are present (at 434, 458, 572, 639, and 747 cm^−1^), while for the other sorbents (fully amorphous), only two bands are present (at 595 and 700 cm^−1^). Taking into account previous papers, Ortíz-Oliveros et al. [50] comprehensively summarized the attribution of the observed bands in this region; the band at 741 cm^−1^ is attributed to the out-of-plane bending mode of the P-OH group, the bands at 750 and 650 cm^−1^ in the crystalline products are attributed to the non-bridged vibrations of the terminal Ti-O group in the Ti-O-P matrix, the bands at 741 and 613 cm^−1^ can be attributed to the P-OH group and the overlap of the Ti-O group, and the bands at 613 and 495 cm^−1^ are symmetrical bending vibration modes observed for P-O, which are attributed to the double degeneration of PO_4_^3−^ [50]. In view of the foregoing, it may be concluded that in the synthesized materials, all bands are slightly shifted, but several bands may probably be attributed to the subsequent vibrations; the bands at 639 and 747 cm^−1^ in the case of TiP3 and TiP4 materials are probably associated with non-bridged vibrations of the Ti-O terminal group in the Ti-O-P matrix, and the bands with a maximum at 434 and 458 cm^−1^ can probably be attributed to the symmetrical bending vibration modes for P-O bonds. It should be added that in their work, Ortíz-Oliveros et al. [50], studied the crystalline material α-Ti(HPO_4_)_2_∙H_2_O; hence, it can be concluded that samples TiP4 and TiP5 probably contain an alpha phase (α-Ti(HPO_4_)_2_∙H_2_O) or another similar layered structure.

Moderately intensive wide bands in the stretching of the hydroxyl group (O-H) region (2600–3700 cm^−1^) are also observed in all the ZrP samples. It should be noted that in this region, several bands are superimposed, the relative intensity of which varies for samples synthesized at different P:Zr molar ratios in the reaction mixture. The band with a maximum at 3360 cm^−1^ is most intense for samples synthesized at a lower P:Zr molar ratio (ZrP1 and ZrP2) and less intense for materials synthesized at the highest P:Zr molar ratio (ZrP5). Such a band may be associated with the presence of OH groups (not derived from acid phosphate residues) in the material. The band with a maximum at 3140 cm^−1^ is the relatively most intense in the ZrP4 and ZrP5 samples and is probably related to PO-H stretching. The band at 1625 cm^−1^ associated with the presence of water (bending of H-O-H) is present in all the samples. In the P-O stretching region, several differences are visible for the materials obtained. A single main band at 1000 cm^−1^ is observed for the materials synthesized at a lower P:Zr molar ratio in the reaction mixture (ZrP1 and ZrP2); in addition, there are no bands associated with P-O-H bending vibrations in the range of 1100–1200 cm^−1^ (or they are very weak and indistinguishable). For amorphous materials synthesized at a moderate P:Zr molar ratio (ZrP3 and ZrTiP), a strong band at 996–1005 cm^−1^ (probably ν(P-O) symmetric) and a weak shoulder band at 1216–1228 cm^−1^ (probably P-O-H bending vibrations) are observed. For partially crystalline materials synthesized at moderate and high P:Zr molar ratios (ZrP4 and ZrP5), two strong bands with maxima at 949–967 cm^−1^ (probably ν(P-OH) antisymmetric and symmetric) and 1020–1070 cm^−1^ (probably ν(P-O) symmetric with a small antisymmetric component) and a weak shoulder band with a maximum at 1216–1250 cm^−1^ (probably P-O-H bending vibrations) are observed. The attribution of the bands was carried out using data from the literature [79]. The difference in the appearance of the superimposed bands between completely amorphous and partially crystalline materials (the separation of the absorption bands originating from these groups) indicates that, in the case of partially crystalline materials, there are domains in which the surroundings of the phosphate groups are better defined, and there are other types of functional groups than in the case of amorphous materials, where the surroundings of the groups are more uniform.

Weak and indistinguishable bands in the low wavenumbers region (400–820 cm^−1^) are observed for materials synthesized at low P:Zr molar ratios (ZrP1 and ZrP2). Two bands of moderate intensity are observed in this region for the other zirconium materials, at about 600 cm^−1^ and 510 cm^−1^, while for ZrTiP, an additional wide and weak band is observed at 750 cm^−1^, which can be attributed to Ti-O terminal groups in the Ti-O-P matrix [50]. It is important to note that the band at 510 cm^−1^ is the most intense for ZrP5. After a closer look, it seems that the band at 510 cm^−1^ is also composed of several components that have a different relative intensity for materials synthesized at different P:Zr molar ratios in the reaction mixture; for ZrP5, the band is slightly shifted towards lower wavenumbers.

The wide band associated with stretching vibrations of the hydroxyl groups (2600–3500 cm^−1^) varies with the applied P:Ce molar ratio in the reaction mixture for CeP materials. In the case of CeP1 (lowest P:Ce molar ratio, crystalline material), the band is most intense and extremely shifted towards high wavenumbers, while in the case of CeP5 (highest P:Ce molar ratio, amorphous), the band is the least intense and shifted to the lowest wavenumbers. In the case of crystalline materials CeP1, CeP2, and CeP3, the band is composed of at least three superimposed components with the maxima at 3560–3580 cm^−1^ (the narrowest), 3350–3443 cm^−1^ (the most intense), and 3176–3256 cm^−1^ (shoulder band). In the case of amorphous materials, the band associated with O-H valence vibrations is also composed of three components, but shifted to lower wavenumbers and significantly wider. The maxima of such bands are observed at 3320–3330 cm^−1^, 3140–3160 cm^−1^ (main band), and 2820–2920 cm^−1^ (shoulder). Moreover, in the case of amorphous samples, an additional band is observed at 2200–2500 cm^−1^. Deconvolution is needed for a detailed analysis of the superimposed bands, but even from looking at the raw data, it is clear that the band arrangement strongly depends on the synthesis conditions and may be crucial for particular groups containing acidic protons. The residual water band (1620 cm^−1^) is visible in all the materials. In the range characteristic of stretching vibrations related to phosphate groups (780–1280 cm^−1^), large differences are visible. It can be assumed that the band consists of at least three components (with maxima at 935, 991, and 1043–1064 cm^−1^), the intensity of which is different for individual materials, which may correspond to the composition of the surface groups. The band with a maximum at about 935 cm^−1^ is most intense for CeP1 and CeP2, slightly less for CeP3, and for CeP4 and CeP5, the band splits into two less intense bands with maxima at 912 and 947 cm^−1^. The band with a maximum at 991 cm^−1^ is less intensive in the case of CeP1 and CeP2, for CeP3, it is distinct and slightly shifted to 989 cm^−1^, while for CeP4 and CeP5, it is shifted to 970 cm^−1^ and is the most intense in this group of bands. The band with a maximum at 1043–1064 cm^−1^ exhibits relatively the lowest intensity (in this group of bands) for CeP1, CeP2, and CeP3, while in the case of CeP4 and CeP5, it is almost as intense as the bands in the range previously discussed. Such results clearly correspond to the differences observed in the range of OH valence bands. Thus, with the increase in the P:Ce molar ratio in the reaction mixture, not only is the transition from a crystalline to an amorphous structure observed, but also the composition of phosphate groups changes, which is very intuitive. According to the literature, the band with a maximum at 1064 cm^−1^ can probably be assigned to the antisymmetric stretching ν(P-O), while the bands with maxima at 935 and 991 cm^−1^ are probably symmetric ν(P-O), superimposed with symmetric and antisymmetric ν(P-OH) [80]. It is known from the literature that the antisymmetric stretching of hydrogen phosphates and phosphates is superimposed and occurs at a higher wavenumber, while the symmetric stretching of phosphate groups occurs slightly below 1000 cm^−1^ for many phosphates [50,79,80]. The bands in the O-P-O bending region (590–680 cm^−1^) are also different for the materials obtained. Two bands appear to be superimposed, with maxima at 630–638 cm^−1^ (most intense for CeP1) and 619 cm^−1^ (most intense for CeP5). This region also corresponds to the previously discussed trends.

Comparing the FTIR spectra for all materials, some general trends can be observed:

- In all cases, the broad band associated with O-H valence vibrations consists of at least three components, the intensity and position of which depend on the P:Metal(IV) molar ratio in the reaction mixture and the degree of crystallinity of the material. The observed band is narrower and shifted towards higher wavenumbers, and contains a characteristic narrow component at around 3500–3600 cm^−1^ for more crystalline materials, while for more amorphous materials, the band is wider and shifted to lower wavenumbers;
- The complex band associated with the stretching vibration of P-O moieties and P-O-H moieties (800–1200 cm^−1^) also depends on the degree of crystallinity and P:Metal(IV) molar ratio applied in the synthesis. At first glance, the trend in the case of CeP sorbents is the opposite of that for TiP and ZrP sorbents; as the P:Metal(IV) molar ratio increases, the relative intensity of the band with the maximum at lower wavenumbers (935–967 cm^−1^) increases for TiP and ZrP sorbents, and decreases for CeP sorbents. However, taking into account the degree of crystallinity of the materials, the trends for all phosphate sorbents are the same—for crystalline materials, the relative intensity of the band with a maximum at 935–967 cm^−1^ increases. This trend is also observed in the case of bending bands in the region below 900 cm^−1^; for samples with a higher degree of crystallinity, the relative intensity of the band shifted to higher wavenumbers increases. Furthermore, in some cases, the shape of such bands is also different; for amorphous materials, the bands are wider, which is very intuitive, and the frequency of the observed vibrations is slightly and randomly disturbed by the undefined surroundings of the functional groups.

### 3.2. Sorption Properties of Titanium(IV), Zirconium(IV), and Cerium(IV) Phosphate Sorbents in Pure Copper Ions Solution—Study of Sorption Isotherms and Kinetics

**Figure 4:**
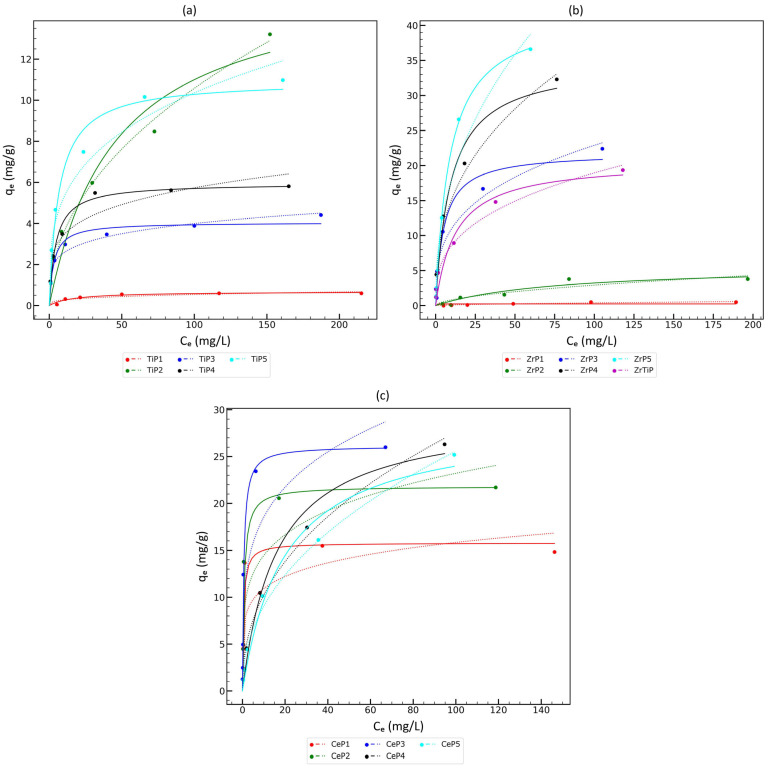
The sorption isotherms for: (**a**) titanium(IV) phosphate sorbents; (**b**) zirconium(IV) phosphate sorbents; and (**c**) cerium(IV) phosphate sorbents. The best-fitted Langmuir model is marked with a solid line, while the Freundlich model is marked with a dotted line.

According to data in the literature, metal(IV) phosphates, mainly titanium(IV) phosphates, are considered promising materials for copper removal from wastewater, especially that of mine origin [67,68]. At first, sorption isotherms were studied for pure diluted copper sulfate solutions. The aim of this stage of the study was to determine the sorption parameters under optimal conditions. Since the sorption of copper ions is important only for low concentrations (for higher concentrations, cementation is the typical method), sorption tests were performed in the concentration range up to 200 mg/L. Many isotherm models are often used in sorption studies, but two of the most important ones (the Langmuir model and the Freundlich model) were used in our experiment. The sorption isotherms for TiP, ZrP, and CeP materials are presented in Figure 4. The fitting parameters of the Langmuir and Freundlich isotherm models are presented in Table 6.

Titanium(IV) phosphates exhibit the lowest ion-exchange capacity for copper in comparison with CeP and ZrP materials. Also, in the case of TiP materials, there are some differences in the character of the sorption isotherms: for almost all materials, the Langmuir model is a better fit (higher *R^2^* values) than the Freundlich model; only the TiP2 material is better described by the Freundlich model. Moreover, this material has an extremely high ion-exchange capacity compared to other TiP materials. If we take a series of materials with an increasing P:Ti molar ratio, i.e., TiP1, TiP3, TiP4, and TiP5, it can be seen that the ion-exchange capacity (*q*_m_) increases monotonically. The exception is the completely amorphous material TiP2. The last difference between TiP2 and other titanium phosphates is the lowest Langmuir isotherm constant *K_L_*, which is about 10 times lower in comparison to TiP3, TiP4, and TiP5, and is even lower than *K_L_* for TiP1, which shows the worst sorption properties. Comparing the ion-exchange capacity for copper (converted to meq/g units) with the Na^+^ uptake determined as a result of the titration experiment, it can be noted that only 0.2–3.4% of the available adsorption centers are occupied, so the copper sorption on the titanium phosphate will be limited only by the Langmuir isotherm constant *K_L_*, which is a measure of the affinity of the material to copper ions.

Zirconium(IV) phosphates exhibit an ion-exchange capacity for copper comparable to cerium phosphates for materials synthesized in excess of phosphoric acid (P:Metal(IV) molar ratio in the reaction mixture > 1) with one exception, i.e., ZrP5, which has a *q_m_* about two times higher than other materials. It should be mentioned that the adsorption constant of ZrP5 is comparable to that of other well-sorbing materials, such as ZrP4 and ZrP3. Zirconium phosphate sorbents are generally better described by the Langmuir model than the Freundlich model. Mixed zirconium titanium phosphate is characterized by an ion-exchange capacity similar to that of ZrP4 and is better described by the Freundlich model, but this material is non-homogeneous (Table 6). The ion-exchange capacity of copper (converted into meq/g) and the Na^+^ ions uptake established as a result of the titration experiment indicate that the sorption of copper by zirconium phosphates is limited only by the Langmuir isotherm constant *K_L_*, which is a measure of the affinity of the sorbent for copper ions, due to the fact that only 0.5–15% of the available adsorption centers are occupied.

The difference in the isotherm plot for crystalline and amorphous cerium phosphates is significant. Only the Langmuir model is well fitted for crystalline materials (CeP1, CeP2, and CeP3), while for amorphous materials, both models are well fitted (even though the Freundlich model may be considered a better model). Moreover, the Langmuir constant *K_L_* is about 30 times higher for crystalline materials than for amorphous materials, while the *q_m_* is slightly lower for crystalline materials (about 70% of the *q_m_* in amorphous materials). Thus, it may be concluded that crystalline cerium phosphates have a higher affinity for copper at lower concentrations. The exponent *n* in the Freundlich equation is higher than 1 in all the cases, so the copper ions probably interact with more than one number of active sites on the sorbent surface. The ion-exchange capacity of copper (converted into meq/g) and the Na^+^ uptake established as a result of the titration experiment indicate that the sorption of copper by cerium phosphate is only limited by the Langmuir constant *K_L_*, which is a measure of the affinity of the sorbent to copper ions, due to the fact that only 12–20% of the available adsorption centers are occupied.

**Figure 5:**
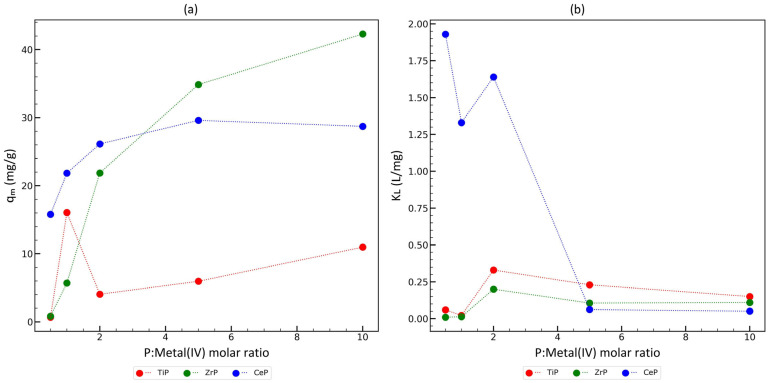
Correlation between: (**a**) *q_m_* and P:Metal(IV) molar ratio in the reaction mixture; (**b**) *K_L_* and P:Metal(IV) molar ratio in the reaction mixture.

Analyzing the sorption isotherms, several general observations can be made:

- The Langmuir model better describes materials which have a crystalline structure or contain some crystalline domains (even poorly formed ones), while the Freundlich model is better in the cases of completely amorphous materials. This observation is consistent with the fact that the Langmuir model is very simple and predicts a high homogeneity of the sorbent surface (the Langmuir model is a so-called local isotherm with a single adsorption energy, so it is assumed that all adsorption centers are equivalent). The Freundlich model is also a simple formula, but it is empirical and has no theoretical justification. However, the shape of the curve is more similar to the so-called generalized Langmuir isotherm, which is a global isotherm model that takes into account the heterogeneity of adsorption energy for different adsorption centers on the material surface. Thus, it can be assumed that completely amorphous materials have a wide range of adsorption centers characterized by different adsorption energies. Detailed physicochemical considerations were not the aim of this article; hence, we only described our observations qualitatively;
- Materials synthesized at a P:Metal(IV) molar ratio higher than 1 show a higher sorption capacity in all the cases (ZrP sorbents: 20–42 mg Cu/g, CeP sorbents: 26–29 mg Cu/g, TiP sorbents: 4–16 mg Cu/g). Furthermore, some linear correlation between the *q_m_* and P:Metal(IV) molar ratio in the reaction mixture can be drawn (Figure 5a), but it is also clearly visible that there are some outlier points;
- The maximum sorption capacity of copper ions (*q_m_*) does not correlate with the Na^+^ uptake, which is the highest for TiP sorbents and the lowest for CeP sorbents, so the sorption capacity for copper ions is not limited by the concentration of acidic ion-exchange surface groups. The Langmuir constant *K_L_* for materials showing a high sorption capacity of copper ions (arbitrarily assumed *q_m_* > 10 mg Cu/g) is within a very wide range of values: ZrP sorbents: 0.08–0.2 L/mg, CeP sorbents: 0.05–1.93 L/mg, and TiP sorbents: 0.02–0.15 L/mg. Therefore, the affinity for copper ions at a low concentration may probably be tuned by the careful selection of synthesis parameters. In addition, there is most likely an optimal P:Metal(IV) molar ratio for the synthesis of the material (Figure 5b).

Sorption kinetics are of great practical importance; therefore, kinetic experiments were performed for each sorbent in order to initially evaluate the functional properties of the obtained materials. It turned out that, for most phosphate sorbents, the process takes place within a few minutes (90% of equilibrium capacity was reached in less than 7 min), which is a great advantage of the obtained materials. Such high process rates make it possible to run the process continuously at high flow rates. According to the literature, the sorption of copper ions using various materials is typically slower, and the time after which 90% of the equilibrium capacity is reached ranges from 20–30 min to even several hours [81,82,83,84]. Due to the high sorption rates, the obtained results are presented in the form of a table containing the time after which a given sorbent achieved 90% of its equilibrium capacity in a solution with a specified concentration of copper ions (Table 7).

Analyzing the table, the following differences between the materials can be pointed out:

- Sorption is the fastest in the case of titanium materials and the slowest in the case of cerium materials;
- Equilibrium is reached more slowly for experiments conducted at higher initial copper ions concentrations, which is associated with the fact that the process is limited only by the availability of active sorption centers in the sorbent, and not by the concentration of ions in the solution (which is typical for sorption processes);
- In the case of cerium materials, the process rate increases significantly with the increase in the P:Metal(IV) molar ratio, which is probably associated with a decrease in the content of the crystalline phase in the material. This tendency probably also occurs in the case of titanium and zirconium materials, but in the tested concentration range, the sorption runs too fast to notice such an effect.

The results obtained in this work are consistent with the data in the literature. High sorption rates were observed for amorphous materials based on titanium phosphate (the time after which 90% of the equilibrium capacity is reached ranged from 5 to 10 min) [45,68], while for crystalline materials, the equilibrium was reached in a much longer period of time (e.g., 1000 min for sorption of Eu on α-TiP [50]).

### 3.3. Sorption Properties of Titanium(IV), Zirconium(IV), and Cerium(IV) Phosphate Sorbents in Solutions of Increasing Complexity—Comparative Studies Using Commercial Ion-Exchange Resins

To evaluate the selectivity of copper capture, distribution coefficients were determined in four solutions of increasing complexity. Ranges of the individual ions concentrations used (Table 2) and the pH of the solution (adjusted to a value of about 2 in equilibrium state) were selected to be close to the concentrations and pH in real acidic mine-drainage wastewater. The use of four model solutions allows us to assess which parameters of the tested solution affect the sorption properties of the obtained sorbents.

The two best materials from each group (TiP2, TiP5, ZrP4, ZrP5, CeP3, and CeP4) and a mixed material ZrTiP were selected for the experiment. Seven commercially available ion-exchange resins (Puromet^MT^ MTS9100, Puromet^MT^ MTS9300, Puromet^MT^ MTS9500, AmberLite^TM^ HPR1200 H, AmberSep^TM^ M4195, LEWATIT^®^ MonoPlus TP 260, and DIAION^TM^ CR20) widely used in heavy metal capture were also included in the experiment. The obtained results are presented as the decimal logarithm of the distribution coefficient (*log K_d_*) (Table 8), due to the wide range of values of the obtained *K_d_* (from 7 to about 45,000 mL/g). In addition, for the most complex solution, the separation factors *SF*_*Cu*/*Me*_ were calculated for the copper capture over the capture of other heavy metals (Table 9).

CeP3 and ZrP5 sorbents show the highest affinity for copper ions (*log K_d_* > 4) in a pure solution of 20 mg/L (Solution 1). The *log K_d_* for the other synthesized materials ranges from 2.6 to 3.9. Commercially available resins reach *log K_d_* values ranging from 0.8 (CR20) to 3.9 (HPR1200H). To sum up, in a pure solution, phosphate materials exhibit comparable or even better affinities than state-of-the-art commercially available materials.

The addition of 1000 mg/L of sodium and potassium ions to Solution 1 significantly reduces the affinity for copper ions by a value ranging from 0.9 to 3.1 on a logarithmic scale for the synthesized materials. The greatest effect is observed for CeP materials. Therefore, it may be concluded that CeP sorbents should be considered as general-purpose ion exchangers rather than copper-selective materials. The *log K_d_* for phosphate sorbents is in the range of 1.6–2.4, while the *log K_d_* for resins is in the range of 1.1–3.0, so phosphate sorbents have a comparable affinity for copper ions in a solution with a salinity typical for brackish waters. Zirconium materials have the best properties (*log K_d_* = 2.3–2.4) among the synthesized materials, and are better than CR20, comparable with MTS9100 and MTS9500, and worse than HPR1200H, TP260, M4195, and MTS9300. It should be noted that for the resins tested, the effect of sodium and potassium ions on the affinity for copper ions is minimal.

The addition of 200 mg/L of magnesium and 20 mg/L of calcium ions (saturated sulfate solution of calcium ions) to Solution 2 has a relatively small effect (decrease in *log K_d_* by 0–0.3) on the affinity for copper ions in the case of phosphate materials, while in the case of some resins (HPR1200H and TP260), the effect is significant. Here, it should be mentioned that the iminodiacetic resin MTS9300 achieves better results than in solutions of lower complexity (Solution 1 and Solution 2). Zirconium materials have the best properties under these conditions (*log K_d_* = 2.1–2.2) among the synthesized materials and are better than CR20 and HPR1200H, comparable with MTS9500, and worse than MTS9100, TP260, M4195, and MTS9300.

The addition of heavy metal ions to Solution 3 at a concentration comparable to that of copper ions (20 mg/L) has a small effect on the affinity for copper ions for both phosphate materials and commercial resins. In some cases, even the observed affinities for copper ions are slightly better (e.g., ZrP5). Also in this test, zirconium materials have the best properties (*log K_d_* = 2.3–2.5) among the synthesized materials and are better than CR20, HPR1200H, and MTS9500, comparable with TP260 and MTS9100, and worse than M4195 and MTS9300. Therefore, the obtained zirconium materials can compete with commercially available resins in terms of their selectivity for copper capture from acidic solutions. Considering the simple production process and relatively low cost, these materials may be an interesting alternative to ion-exchange resins.

In some cases, the selectivity of copper binding in comparison with other heavy metals is significant; therefore, the separation factors (Table 9) were analyzed. Zirconium materials in all cases exhibit separation factors higher than 1, indicating a preference for copper capture from complex solutions. Titanium materials, mixed zirconium titanium material, and cerium material CeP3 can be considered promising sorbents for the separation of copper from manganese.

The selectivity of copper over against cadmium for zirconium phosphate sorbents is higher than for HPR1200H, M4195, MTS9500, and TP260, and significantly lower than for MTS9100 and MTS9300. When comparing copper and nickel sorption, zirconium materials show a better selectivity towards copper in relation to nickel than HPR1200H and M4195, and significantly worse than TP260, MTS9500, and CR20 resins. Analyzing the values of the separation factor of copper in relation to manganese, it can be seen that zirconium sorbents are characterized by a better copper separation than MTS9500 and TP260 resins, and considerably worse than other resins. For the separation of copper from zinc, zirconium sorbents perform better than HPR1200H and TP260 resins, and much worse than MTS9300 and CR20 resins. The preferential sorption of copper over iron occurs more efficiently in the case of zirconium phosphate sorbents than CR20, HPR1200H, MTS9500, and TP260 resins.

Taking into account the affinity to the copper ions and the selectivity towards all tested ions, it can be concluded that the best phosphate material ZrP5 exhibits better properties (with some exceptions) than CR20, HPR1200H, MTS9500, and TP260 under the tested conditions. M4195 and MTS9300 resins show significantly better properties in copper sorption than ZrP5, both in terms of their affinity and selectivity, while MTS9100 resin is characterized by a higher selectivity but lower affinity.

## 4. Conclusions

Sixteen metal(IV) phosphate sorbents (titanium, zirconium, and cerium) were successfully synthesized under mild and scalable conditions, and characterized. The structural and functional properties of the obtained materials were compared, and the observed trends were discussed in detail. In addition, the sorption properties of the obtained phosphate sorbents were compared with those of commercially available ion-exchange resins.

An important parameter of the synthesis of phosphate materials that was analyzed is the P:Metal(IV) molar ratio in the reaction mixture. Materials with the best sorption properties were obtained at a P:Metal(IV) molar ratio of 1:1 and higher. The sorption properties of tetravalent metal phosphates were tested in relation to copper ions. High ion-exchange capacities were achieved for the best materials in each group: 16.9 meq/g for TiP sorbents, 8.8 meq/g for ZrP sorbents, and 7.0 meq/g for CeP sorbents. Such values are several times higher than the ion-exchange capacities for commercial resins (≈3.2 meq/g), so obtained materials may be considered a low-cost alternative to general-purpose polymeric ion-exchange resins. Moreover, the resulting sorbents exhibit good kinetic properties. For all the titanium sorbents and most zirconium sorbents, the sorption equilibrium was reached within about 7 min, regardless of the initial copper ions concentration in the solution. Therefore, the obtained phosphate materials may be used in dynamic systems (e.g., sorption columns).

For the best sorbents, the affinity and selectivity in relation to the capture of copper ions from complex acidic solutions (pH of about 2) were evaluated and compared to commercially available resins (containing various surface functional groups) intended for copper removal. Based on the obtained results, it was found that the most promising sorbent is zirconium phosphate synthesized at a P:Zr molar ratio in the reaction mixture of 10:1. This material exhibits the best sorption properties towards copper ions, better than the general-purpose AmberLite^TM^ HPR1200H resin (with sulphonic surface functional groups), aminophosphonic resins (Puromet^MT^ MTS9500 and LEWATIT^®^ MonoPlus TP260), and polyamine-chelating resin Dianion CR20, and comparable with amidoxime-chelating resin Puromet^MT^ MTS9100 and worse than bis-picolylamine resin AmberSep^TM^ M4195 and iminodiacetic resin Puromet^MT^ MTS9300.

To sum up, our studies show that zirconium phosphate-based materials may compete with chelating resins in some applications (e.g., the treatment of acid mine drainage), where their low cost and simplicity of sorbent preparation will be a key advantage. Furthermore, the trend in material properties we have observed will provide guidance in the further development of this type of materials. To fully evaluate the suitability of the zirconium sorbents for copper removal from acid wastewater of mine origin, additional studies are needed. Therefore, experiments in dynamic systems as well as regeneration studies will be the subject of future work.

## Figures and Tables

**Table 1:** Characteristics of the tested ion-exchange resins [69,70,71,72,73,74,75].

Resin Name	Type of Resin	Type of Resin Structure	Appearance	Polymer Structure	Functional Group	Ionic Form	Exchange Capacity	Main Application
Puromet^MT^ MTS9100	chelating	macroporous	beige spherical beads (300–1200 μm)	polyacrylic-divinylbenzene	amidoxime	free base	40 g/L (Cu)	recovery of precious metals
Puromet^MT^ MTS9300	chelating	macroporous	beige spherical beads (425–1000 μm)	polystyrene-divinylbenzene	iminodiacetic	Na^+^	50 g/L (Cu)	removal of Cu, Co, Ni, and Zn
Puromet^MT^ MTS9500	chelating	macroporous	beige spherical beads (300–1200 μm)	polystyrene-divinylbenzene	aminophosphonic	Na^+^	26 g/L (Ca)	removal of Fe, Cu, Zn, Sb, and Bi
AmberLite^TM^ HPR1200 H	strong acid cation	gel	dark-brown spherical beds (600 ± 50 μm)	styrene-divinylbenzene	sulfonic	H^+^	≥1.8 eq/L (H^+^ form)	industrial demineralization
AmberSep^TM^ M4195	chelating	macroporous	tan to dark-brown to dark-green spherical beads (297–841 μm)	styrene-divinylbenzene	bis-picolylamine	weak base/ partial H_2_SO_4_ salt	≥35 g/L (Cu)	recovery of Cu and Ni
DIAION^TM^ CR20	chelating	highly porous	light-yellow spherical beads (300–1180 μm)	styrene-divinylbenzene	polyamine	free base	≥0.4 mol/L (Cu)	removal of metal cations from wastewater
LEWATIT^®^ MonoPlus TP 260	chelating	macroporous	beige spherical beads (630 ± 50 μm)	styrene-divinylbenzene	aminomethylphosphonic	Na^+^	≥2.4 eq/L (H^+^ form)	removal of heavy metal and alkaline earth cations

**Table 2:** The compositions of the solutions used in the comparative study.

Metal (mg/L)	Solution 1	Solution 2	Solution 3	Solution 4
Cu^2+^	20	20	20	20
Na^+^	-	1000	1000	1000
K^+^	-	1000	1000	1000
Mg^2+^	-	-	200	200
Ca^2+^	-	-	25	25
Cd^2+^	-	-	-	20
Mn^2+^	-	-	-	20
Ni^2+^	-	-	-	20
Zn^2+^	-	-	-	20
Fe^3+^	-	-	-	10

**Table 3:** Data on the synthesis of tetravalent metal phosphates.

Synthesis of Titanium(IV) Phosphates			
Sorbent Name	P Source	Ti Source	P:Ti Molar Ratio
TiP1	85% H_3_PO_4_	titanium(IV) oxide sulfate sulfuric acid hydrate	0.5:1
TiP2			1:1
TiP3			2:1
TiP4			5:1
TiP5			10:1
**Synthesis of Zirconium(IV) Phosphates**			
**Sorbent Name**	**P Source**	**Zr Source**	**P:Zr Molar Ratio**
ZrP1	42.5% H_3_PO_4_	zirconium carbonate basic hydrate reacted with 65% HNO_3_	0.5:1
ZrP2			1:1
ZrP3			2:1
ZrP4			5:1
ZrP5			10:1
**Synthesis of Zirconium Titanium Phosphate**			
**Sorbent Name**	**P Source**	**Zr and Ti Source**	**P:(Zr+Ti) Molar Ratio**
ZrTiP	42.5% H_3_PO_4_	zirconium carbonate basic hydrate reacted with 65% HNO_3_ titanium(IV) oxide sulfate sulfuric acid hydrate	2:1
**Synthesis of Cerium(IV) Phosphates**			
**Sorbent Name**	**P Source**	**Ce Source**	**P:Ce Molar Ratio**
CeP1	42.5% H_3_PO_4_	cerium(IV) phosphate tetrahydrate	0.5:1
CeP2			1:1
CeP3			2:1
CeP4			5:1
CeP5			10:1

**Table 4:** Composition of metal(IV) phosphate sorbents determined based on SEM-EDS analysis.

Sorbent Name	Metal(IV) [Atomic %]			P [Atomic %]		O [Atomic %]		P:Metal(IV) Molar Ratio ^b^
	Metal(IV)	Mean	Range ^a^	Mean	Range ^a^	Mean	Range ^a^	
TiP1	Ti	15.2	8.2	10.3	3.5	74.5	11.0	0.68 (0.56–0.75)
TiP2		13.1	4.4	11.8	3.8	75.1	8.3	0.90 (0.86–0.91)
TiP3		10.3	3.9	11.5	5.2	78.2	9.0	1.12 (1.02–1.23)
TiP4		13.7	3.3	14.4	5.8	71.9	8.7	1.05 (0.90–1.13)
TiP5		10.3	1.4	12.4	1.8	77.3	3.2	1.20 (1.10–1.27)
ZrP1	Zr	15.9	4.8	6.9	4.0	77.2	6.4	0.43 (0.34–0.57)
ZrP2		20.1	16.1	12.5	9.8	67.4	25.9	0.62 (0.57–0.70)
ZrP3		13.5	14.0	13.4	14.0	73.1	27.8	0.99 (0.78–1.21)
ZrP4		9.1	4.1	14.1	5.6	76.8	10.6	1.55 (1.08–1.88)
ZrP5		8.8	4.0	14.4	3.3	76.8	7.2	1.64 (1.43–1.89)
ZrTiP	Zr	11.4	13.0	15.8	16.8	65.5	37.2	0.84 ^c^ (0.72–1.12)
	Ti	7.3	9.0					
CeP1	Ce	12.4	0.6	14.2	0.2	73.4	0.8	1.15 (1.12–1.16)
CeP2		11.2	1.0	14.1	1.8	74.7	2.9	1.26 (1.21–1.30)
CeP3		12.4	2.4	16.2	2.9	71.4	5.3	1.31 (1.25–1.32)
CeP4		10.8	3.7	15.1	3.1	74.1	6.0	1.40 (1.18–1.57)
CeP5		13.5	17.2	17.0	9.9	69.5	27.2	1.26 (0.96–1.77)

^a^ The difference between the maximum and minimum atomic concentration at the analyzed points of the sorbent sample. ^b^ The mean P:Metal(IV) molar ratio was calculated from the SEM-EDS analysis; the range of values from the analysis of individual points is given in brackets. ^c^ P:(Zr+Ti).

**Table 5:** Total ion-exchange capacity for synthesized materials.

Sorbent Name	Ion-Exchange Capacity (meq/g) ^a^	P:Metal(IV) Molar Ratio ^a^	Calculated Formula of the Material ^b^
TiP1	12.3	0.67 (0.56–0.75)	Ti(HPO_4_)_0.67_(OH)_1.16_O_0.75_(H_2_O)_0.28_
TiP2	15.0	0.89 (0.86–0.91)	Ti(HPO_4_)_0.89_(OH)_1.66_O_0.28_(H_2_O)_0.23_
TiP3	15.4	1.12 (1.02–1.23)	Ti(H_2_PO_4_)_0.17_(HPO_4_)_0.95_(OH)_1.93_(H_2_O)_1.18_
TiP4	15.5	1.04 (0.90–1.13)	non-homogeneous material
TiP5	16.9	1.20 (1.10–1.27)	Ti(H_2_PO_4_)_0.38_(HPO_4_)_0.82_(OH)_1.98_(H_2_O)_0.73_
ZrP1	4.9	0.43 (0.34–0.57)	Zr(HPO_4_)_0.43_(OH)_0.48_O_1.33_(H_2_O)_1.3_
ZrP2	5.7	0.62 (0.57–0.70)	non-homogeneous material
ZrP3	6.6	0.99 (0.78–1.21)	non-homogeneous material
ZrP4	8.7	1.55 (1.08–1.88)	Zr(H_2_PO_4_)_0.88_(HPO_4_)_0.67_O_0.89_(H_2_O)_1.35_
ZrP5	8.8	1.64 (1.43–1.89)	Zr(H_2_PO_4_)_0.89_(HPO_4_)_0.75_O_0.8_(H_2_O)_1.36_
ZrTiP	8.4	0.84 (0.72–1.12)	non-homogeneous material
CeP1	3.8	1.15 (1.12–1.16)	Ce(HPO_4_)_1.04_(PO_4_)_0.11_O_0.79_(H_2_O)_0.53_
CeP2	4.1	1.26 (1.21–1.30)	Ce(HPO_4_)_1.18_(PO_4_)_0.08_O_0.70_(H_2_O)_0.93_
CeP3	4.2	1.30 (1.25–1.32)	Ce(HPO_4_)_1.18_(PO_4_)_0.08_O_0.62_
CeP4	7.5	1.40 (1.18–1.57)	Ce(H_2_PO_4_)_0.82_(HPO_4_)_0.58_O_1.01_(H_2_O)_0.25_
CeP5	7.0	1.26 (0.96–1.77)	non-homogeneous material

^a^ Total Na^+^ uptake until pH 13 is reached. ^b^ Calculations were made based on SEM-EDS and Na^+^ uptake results.

**Table 6:** Fitting parameters of the Langmuir and Freundlich sorption isotherm models.

Sorbent Name	Langmuir Isotherm Model			Freundlich Isotherm Model		
	*q_m_* (mg/g)	*K_L_* (L/mg)	*R* ^2^	*n*	*K_F_* ((mg/g)·(L/mg)^1/n^)	*R* ^2^
TiP1	0.68	0.06	0.92	3.43	0.14	0.76
TiP2	16.05	0.02	0.94	2.11	1.20	0.99
TiP3	4.05	0.33	0.94	5.23	1.66	0.95
TiP4	5.96	0.23	0.98	4.50	2.06	0.89
TiP5	10.97	0.15	0.97	3.40	2.67	0.93
ZrP1	0.83	0.01	0.95	1.62	0.02	0.90
ZrP2	5.70	0.01	0.91	1.79	0.23	0.85
ZrP3	21.87	0.20	0.95	3.13	5.25	0.95
ZrP4	20.13	0.44	0.96	3.75	6.43	0.96
ZrP5	42.29	0.11	0.99	2.32	6.63	0.95
ZrTiP	20.63	0.08	0.96	2.89	3.86	0.99
CeP1	15.79	1.93	0.92	6.20	7.53	0.67
CeP2	21.84	1.33	0.91	4.86	9.00	0.76
CeP3	26.14	1.64	0.97	1.64	10.92	0.81
CeP4	29.60	0.06	0.98	2.32	3.80	0.99
CeP5	28.72	0.05	0.98	2.22	3.20	0.99

**Table 7:** Time after which a given sorbent achieved 90% of its equilibrium capacity in a solution with a specified concentration of copper ions.

Sorbent Name	Times After Which 90% of the Equilibrium Capacity Was Achieved (min)					
	5 (mg/L)	10 (mg/L)	20 (mg/L)	50 (mg/L)	100 (mg/L)	200 (mg/L)
TiP1	<7	<7	<7	<7	<7	<7
TiP2	<7	<7	<7	<7	<7	<7
TiP3	<7	<7	<7	<7	<7	<7
TiP4	<7	<7	<7	<7	<7	<7
TiP5	<7	<7	<7	<7	<7	<7
ZrP1	<7	<7	<7	<7	<7	<7
ZrP2	<7	<7	<7	<7	15	>60
ZrP3	<7	<7	<7	15	60	>60
ZrP4	<7	<7	<7	<7	<7	<7
ZrP5	<7	<7	<7	<7	<7	7
ZrTiP	<7	<7	<7	<7	30	60
CeP1	<7	<7	15	60	>60	>60
CeP2	<7	<7	<7	15	>60	>60
CeP3	<7	<7	<7	15	>60	>60
CeP4	<7	7	15	45	60	>60
CeP5	<7	<7	<7	<7	15	60

**Table 8:** The *log K_d_* values for the best phosphate sorbents and selected commercially available resins determined in four solutions of increasing complexity.

Sorbent/Resin Name	*log K_d_*			
	Solution 1	Solution 2	Solution 3	Solution 4
TiP2	2.6	1.7	1.6	1.6
TiP5	3.4	2.0	1.9	1.9
ZrP4	3.8	2.3	2.2	2.3
ZrP5	4.1	2.4	2.1	2.5
ZrTiP	3.9	2.1	2.0	2.1
CeP3	4.7	1.6	1.5	1.3
CeP4	3.1	1.7	1.7	1.7
CR20	0.8	1.1	1.1	1.0
HPR1200H	3.9	2.8	1.9	1.9
M4195	2.6	3.0	2.8	2.8
MTS9100	2.2	2.4	2.3	2.2
MTS9300	3.1	3.0	3.5	3.4
MTS9500	2.4	2.3	2.1	1.9
TP260	2.9	2.8	2.3	2.2

**Table 9:** The separation factors *SF*_*Cu*/*Me*_ for the copper capture over the capture of other heavy metals determined in the most complex solution (Solution 4).

Sorbent/Resin Name	*SF*_*Cu*/*Cd*_	*SF*_*Cu*/*Ni*_	*SF*_*Cu*/*Mn*_	*SF*_*Cu*/*Zn*_	*SF*_*Cu*/*Fe*_
TiP2	8.1	0.8	186.6	4.2	0.2
TiP5	1.3	2.9	657.9	6.4	2.0
ZrP4	4.3	3.5	2.5	5.0	3.2
ZrP5	4.5	5.5	34.0	5.9	5.1
ZrTiP	5.8	3.7	833.3	3.0	1.3
CeP3	2.1	0.7	147.1	4.7	0.5
CeP4	3.2	1.0	3.2	5.7	1.1
CR20	110.3	110.3	110.3	131.1	2.1
HPR1200H	0.6	0.7	833.3	1.9	1.1
M4195	1.9	1.7	123.9	20.7	38.2
MTS9100	1726.2	11.7	1726.2	79.4	57.6
MTS9300	42.2	12.3	22,857.1	406.1	18.8
MTS9500	1.8	787.0	1.8	6.2	0.1
TP260	1.2	1627.9	1.1	3.2	1.3

## Data Availability

The original contributions presented in the study are included in the article, further inquiries can be directed to the corresponding author.
